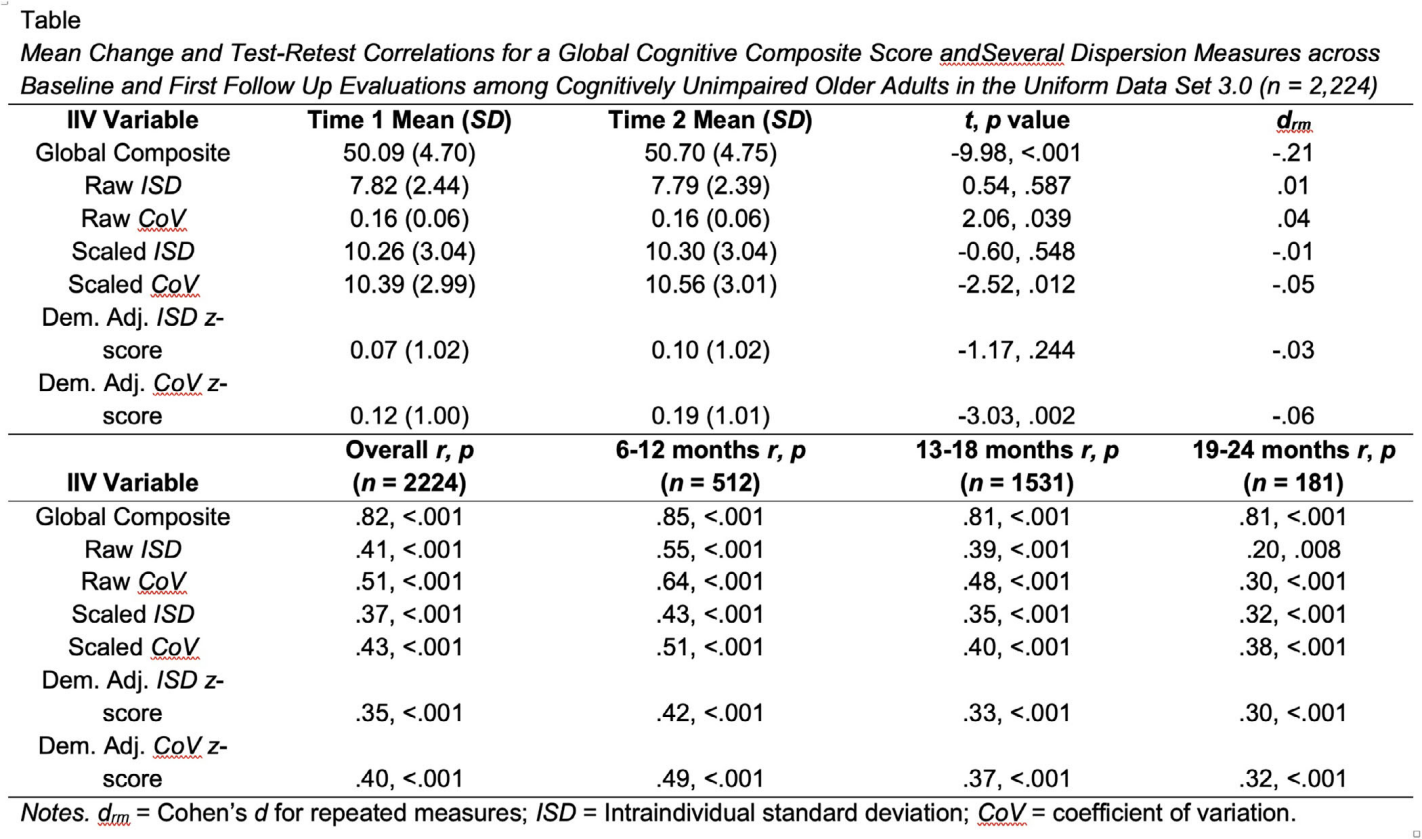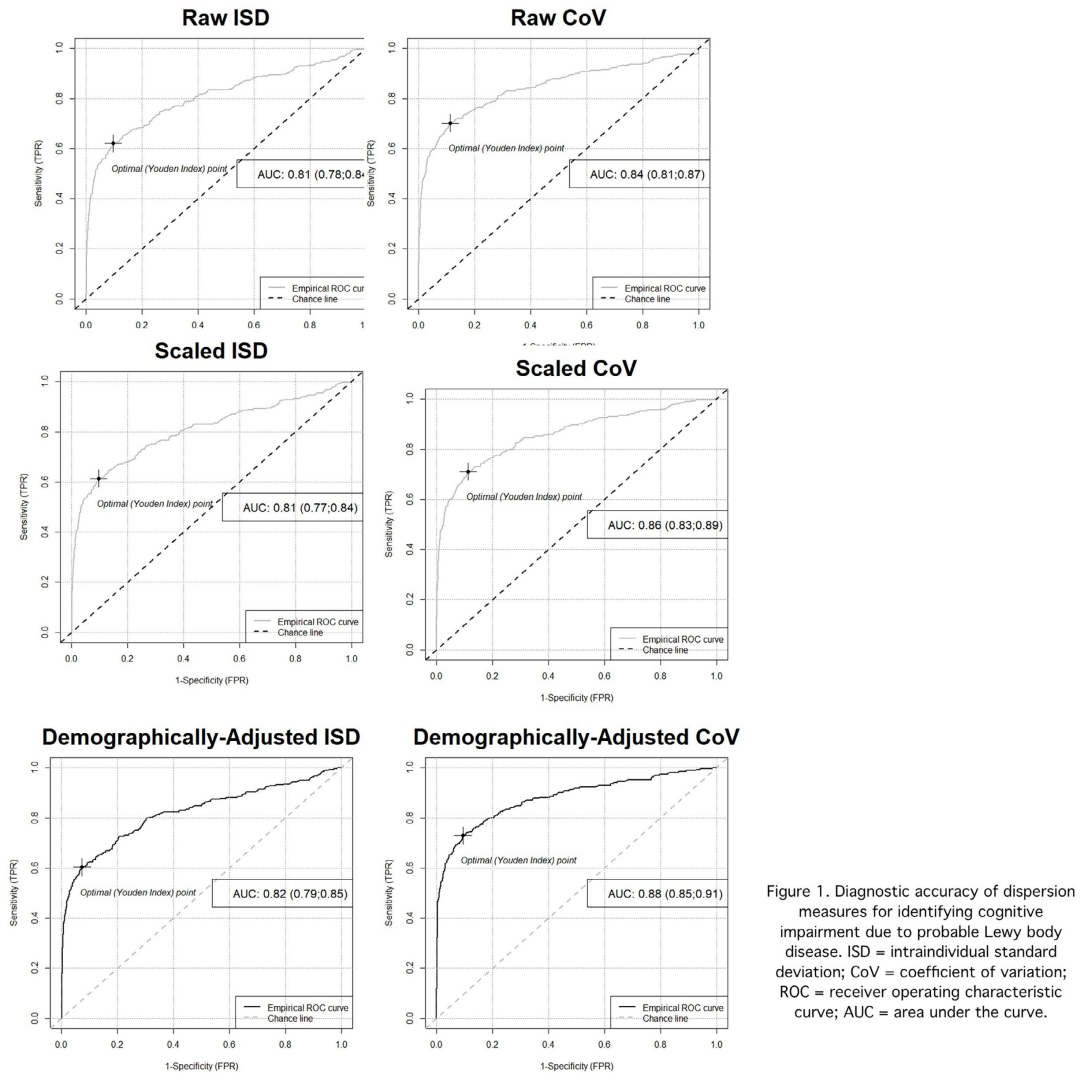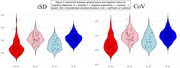# Developing and validating normed scores for cognitive dispersion among older adults

**DOI:** 10.1002/alz70857_105896

**Published:** 2025-12-25

**Authors:** Andrew M. Kiselica, Sidonia Compton, Shayne S.‐H. Lin, Alyssa Kaser, Cynthia Mikula, Alica Milam, Steven P. Woods, Troy Webber, Daniel Weitzner, Stephen Aita, Nicholas Borgnona, Keenan A. Walker, Vidyulata Kamath, Kristina Visscher, Charles F Murchison, David S. Geldmacher, Erik D Roberson, Benjamin D Hill, Anna Boone, Timothy Wolf, Victor Del Bene

**Affiliations:** ^1^ University of Georgia, Athens, GA, USA; ^2^ University of Missouri, Columbia, MO, USA; ^3^ the University of Alabama, Tuscaloosa, AL, USA; ^4^ University of Texas Southwestern Medical Center, Dallas, TX, USA; ^5^ Columbia University, New York, NY, USA; ^6^ Michael E. Debakey VA Hospital, Houston, TX, USA; ^7^ University of Houston, Houston, TX, USA; ^8^ Baycare, Clearwater, FL, USA; ^9^ Maine VA, Augusta, ME, USA; ^10^ University of Alabama Birmingham, Birmingham, AL, USA; ^11^ National Institute of Aging Intramural Research Program, National Institutes of Health, Bethesda, MD, USA; ^12^ Johns Hopkins University School of Medicine, Baltimore, MD, USA; ^13^ University of Alabama at Birmingham, Birmingham, AL, USA; ^14^ University of Alabama, Birmingham, Birmingham, AL, USA; ^15^ Alzheimer's Disease Center, Birmingham, AL, USA; ^16^ University of South Alabama, Mobile, AL, USA

## Abstract

**Background:**

Cognitive dispersion refers to within person variability in performance across a battery of neuropsychological tests. Measures of dispersion are sensitive to severity of cognitive and functional impairment and predictive of biological and clinical disease progression in Alzheimer's disease (AD). Practical application of cognitive dispersion measures has been limited due to lack of tools to support their implementation. We will present three studies leveraging data from the National Alzheimer's Coordinating Center to 1) develop normed score calculation methods for dispersion measures; 2) assess the stability and test‐retest reliability of normed dispersion scores; and 3) examine the relationships of dispersion scores with Alzheimer's disease (AD) biomarkers.

**Method:**

Study 1: We developed demographically adjusted regression‐based normed scores for cognitive dispersion in a sample of 4,283 cognitively unimpaired older adults from the Uniform Data Set 3.0. We then examined the ability of these normed scores to differentiate individuals with cognitive impairment due to Lewy body disease (*n* = 282) from cognitively unimpaired individuals using receiver operating characteristic curves. Study 2: We assessed mean level stability and test‐retest reliability of dispersion scores in a sample of 2,224 robustly cognitively unimpaired older adults from the Uniform Data Set 3.0. Study 3: We utilized SCAN data from 982 older adult participants with AD biomarker testing available to evaluate the relationship between AD biomarker burden and dispersion.

**Result:**

Study 1: We created normative tables and an Excel calculator for deriving normed cognitive dispersion scores. Cognitive dispersion scores differentiated individuals with cognitive impairment due to Lewy body disease from cognitively unimpaired individuals with high accuracy (AUC range = .81‐.88; Figure 1). Study 2: Dispersion scores demonstrated high mean level stability (*d_rm_* range = ‐.06‐.01) but poor test‐retest reliability (*r* range = .35‐.51; Table). Study 3: Across measures of amyloid, tau, and neurodegeneration, increasing AD biomarker burden was related to increasing cognitive dispersion among individuals with cognitive impairment but not among cognitively unimpaired individuals (e.g., Figure 2).

**Conclusion:**

Normed cognitive dispersion scores may have limited test‐retest reliability in cognitively unimpaired older adults; however, they appear sensitive to Lewy body disease clinical features and AD biomarker burden.